# Automated quantification of 3D wound morphology by machine learning and optical coherence tomography in type 2 diabetes

**DOI:** 10.1002/ski2.203

**Published:** 2022-12-21

**Authors:** Yinhai Wang, Adrian Freeman, Ramzi Ajjan, Francesco Del Galdo, Ana Tiganescu

**Affiliations:** ^1^ Data Sciences & Quantitative Biology Discovery Sciences BioPharmaceuticals R&D AstraZeneca Cambridge UK; ^2^ Emerging Innovations Unit Discovery Sciences BioPharmaceuticals R&D AstraZeneca Cambridge UK; ^3^ Leeds Institute of Cardiovascular and Metabolic Medicine University of Leeds Leeds UK; ^4^ NIHR Biomedical Research Centre Leeds Teaching Hospitals NHS Trust Leeds UK; ^5^ Leeds Institute of Rheumatic and Musculoskeletal Medicine University of Leeds Leeds UK

## Abstract

**Background:**

Driven by increased prevalence of type 2 diabetes and ageing populations, wounds affect millions of people each year, but monitoring and treatment remain limited. Glucocorticoid (stress hormones) activation by the enzyme 11β‐hydroxysteroid dehydrogenase type 1 (11β‐HSD1) also impairs healing. We recently reported that 11β‐HSD1 inhibition with oral AZD4017 improves acute wound healing by manual 2D optical coherence tomography (OCT), although this method is subjective and labour‐intensive.

**Objectives:**

Here, we aimed to develop an automated method of 3D OCT for rapid identification and quantification of multiple wound morphologies.

**Methods:**

We analysed 204 3D OCT scans of 3 mm punch biopsies representing 24 480 2D wound image frames. A u‐net method was used for image segmentation into 4 key wound morphologies: early granulation tissue, late granulation tissue, neo‐epidermis, and blood clot. U‐net training was conducted with 0.2% of available frames, with a mini‐batch accuracy of 86%. The trained model was applied to compare segment area (per frame) and volume (per scan) at days 2 and 7 post‐wounding and in AZD4017 compared to placebo.

**Results:**

Automated OCT distinguished wound tissue morphologies, quantifying their volumetric transition during healing, and correlating with corresponding manual measurements. Further, AZD4017 improved epidermal re‐epithelialisation (by manual OCT) with a corresponding trend towards increased neo‐epidermis volume (by automated OCT).

**Conclusion:**

Machine learning and OCT can quantify wound healing for automated, non‐invasive monitoring in real‐time. This sensitive and reproducible new approach offers a step‐change in wound healing research, paving the way for further development in chronic wounds.

## Graphical abstract

Development of novel clinical interventions to improve skin biomechanical properties, for example, AZD4017 for wound healing, is hampered by the lack of reliable objective imaging modalities. Here, we combined machine learning and optical coherence tomography (OCT) to develop an automated wound imaging tool able to quantify multiple acute wound morphologies in 3D. Our novel, automated method (validated against manual 2D wound re‐epithelialisation OCT outputs) was more sensitive, reproducible, objective, and comprehensive than conventional wound imaging. Application of this unbiased and robust imaging tool to clinical trial OCT outputs also corroborated our finding that AZD4017 may improve wound healing in type 2 diabetes.

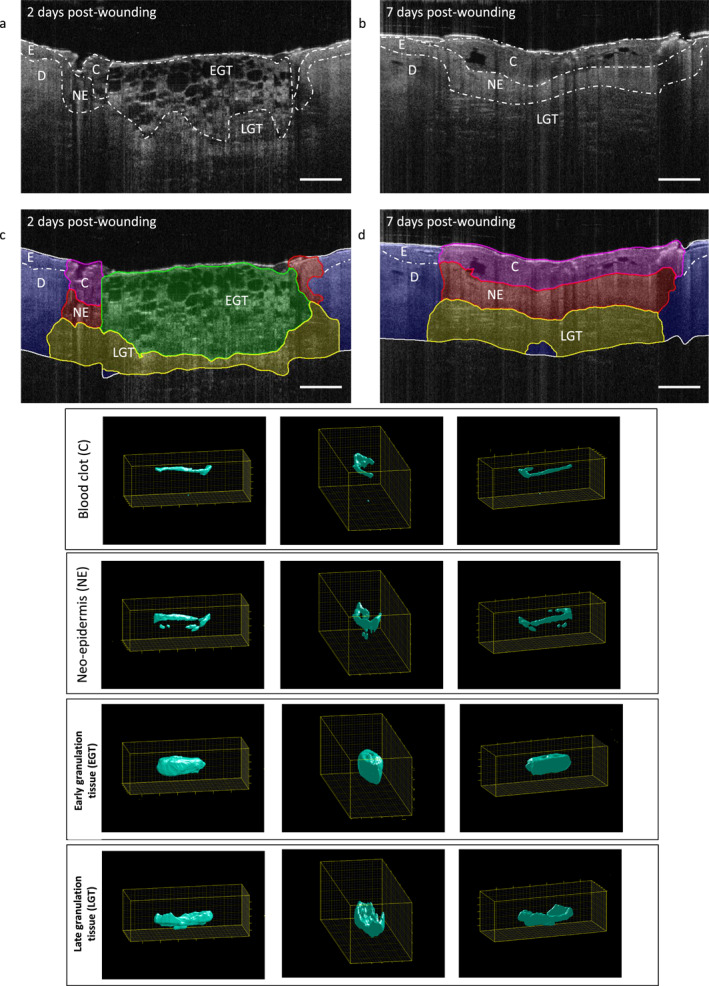

1

**What is already known about this topic?**

- Optical coherence tomography (OCT) imaging offers a biopsy‐free wound healing imaging method.
- OCT image analysis is currently limited to subjective and time‐consuming 2D manual analysis that does not reflect complex wound healing tissue morphology.

**What does this study add?**

- Application of machine learning enabled unbiased, automated quantification of key wound tissue types by OCT in 3D.
- Validation of this sensitive and reproducible wound imaging tool confirmed and strengthened clinical trial OCT findings.
- This approach offers a step‐change in reliability and robustness of OCT as a clinical wound imaging tool, both for research and future development as a companion diagnostic.

## 2 INTRODUCTION

In 2017‐2018, the UK National Health Service treated 3.8 million people with wounds; with one‐third failing to heal within a year.^1^ Direct costs were over £8 billion, and annual wound prevalence increased by over 70% between 2012 and 2018, highlighting the urgent need to improve wound monitoring, management, and treatment.^1^ The financial burden of chronic wounds is mainly attributable to amputations in people with type 2 diabetes mellitus (T2DM).^2^ Since 1980, global rates of T2DM have more than quadrupled, with half of patients failing to meet recommended care goals.^3^, ^4^

Glucocorticoid (stress hormone) excess also drives wound chronicity,^5^, ^6^ affecting virtually every phase of wound healing, including inflammation^7^, ^8^; re‐epithelialisation^9^, ^10^; granulation tissue formation^10^; and collagen remodelling.^11^, ^12^ Local glucocorticoid levels are regulated by 11β‐hydroxysteroid dehydrogenase (11β‐HSD) isozymes that activate (11β‐HSD1) and deactivate (11β‐HSD2) natural and synthetic corticosteroids. Our recent double‐blind, pilot phase 2b randomised controlled trial in adults with T2DM treated with the selective 11β‐HSD1 inhibitor AZD4017 (AZD) for 35 days found a 48% reduction in day 2 punch biopsy wound width versus placebo (PCB) using optical coherence tomography (OCT).^13^ However, automated 3D imaging solutions for the objective measurement of wound healing in clinical research and practice are currently lacking. Wound tracing methods are subjective, time‐consuming and do not capture detailed wound tissue composition and heterogeneity. This reduces the sensitivity and accuracy of wound healing research and limits identification of hard‐to‐heal wounds, leading to delays in novel treatment development and clinical intervention, and poorer healing outcomes.

OCT is a real‐time tomographic imaging technique that uses low‐intensity infrared light to visualise living tissues. This method enables high‐resolution 2D and 3D cross‐sectional imaging and OCT has been developed as a clinical tool for several dermatological conditions including hypokeratosis,^14^ psoriasis,^15^, ^16^ scleroderma^17^, ^18^ and cancer.^19^, ^20^ OCT imaging has also been validated for skin wound healing.^21^, ^22^, ^23^, ^24^, ^25^, ^26^

An important advantage of OCT compared to photography‐based wound imaging is that the structure of healing tissue can be visualised below the skin surface, potentially providing useful information about the underlying wound bed without invasive biopsies. However, current manual annotation of 2D scans is labour‐intensive, subjective, and limits OCT imaging potential. Hence, an automated approach to OCT tissue quantification using machine learning could enable practical and scalable application of OCT as a more standard modality for studying and monitoring wound healing.

Here, we developed a volumetric OCT machine learning algorithm to identify and quantify key morphological features of wound healing, which also corroborated our recent clinical trial findings towards a novel wound healing therapy.^13^

## 3 METHODS

### 3.1 Study participants

This study was conducted with approval from the North West Greater Manchester Central Research Ethics Committee (17/NW/0283), with study participant written informed consent. Briefly, participants were randomised in a double‐blind manner to PCB (*n* = 14) or 400 mg twice daily of the 11β‐HSD1 inhibitor AZD (*n* = 14) for 35 days. At day 0 (baseline, pre‐treatment), two 3 mm diameter full‐thickness lower outer arm punch biopsies were conducted under local anaesthesia, repeated in the contralateral arm at day 28. Wounds were treated with a breathable dressing for 24 h and imaged on days 2 and 7 post‐wounding (i.e., days 2 and 7 after day 0 biopsies, and days 30 and 35 after day 28 biopsies). Full study details and patient demographics are as previously reported.^13^

### 3.2 Gross morphology and re‐epithelialisation

Gross wound morphology was measured manually from aerial camera images (built‐in to the OCT scanner) using ImageJ (National Institute of Health). Re‐epithelialisation was measured manually from OCT surface images using ImageJ.

### 3.3 Optical coherence tomography

We used a Michelson‐Diagnostics (Maidstone) VivoSight^TM^ scanner to capture 120 image frames 50 μm apart (6 mm) for each 3 mm punch biopsy. A total of 355 scans from 36 participants were conducted with two biopsies at each time point per patient. This data included unwounded skin scans, replicated scans of the same biopsy (e.g., scan failure) and study eligibility screen failures not randomised to AZD or PCB. In a small number of cases only one biopsy was scanned (e.g., due to active bleeding). Three scans were removed prior to machine learning input due to image anomalies (e.g., a single scan capturing two adjoining biopsies). After omitting unwounded skin, replicate scans, screen fails and anomalies, we retained 204 scans (24 480 image frames) representing all 28 randomised study participants.

### 3.4 Machine learning

Within each scan frame, seven distinctive image subtypes were defined: early granulation tissue, late granulation tissue, neo‐epidermis, blood clot, intact tissue (peripheral to the wound), active bleeding, and non‐tissue. We used a u‐net‐based convolutional neural network for image segmentation of each 2D OCT frame, and the ground truth labels were manual annotations of the seven subtypes.^27^ We acknowledge that other networks can also be used,^28^, ^29^ but for this early proof‐of‐concept study, we used classical u‐net architecture. Downstream data analysis after segmentation focussed on wound‐relevant image subtypes that is, early granulation tissue, late granulation tissue, neo‐epidermis, and blood clot that is, non‐tissue, intact tissue and active bleeding (which only appeared in a few cases) were not analysed.

From the original set of 355 OCT scans (each comprising 120 frames), we manually selected 84 frames (0.2%, representing all participants and time points) for u‐net training. Selection followed the principle of enabling the training process to ‘see’ a variety of wound morphologies manually annotated for the seven image subtypes. We acknowledge the existence of various image augmentation methods could be used in the future to further improve training.^30^ U‐net architecture was trained in a Linux cluster with three T K80 graphics processing units, requiring 100 epochs with 1344 iterations per epoch, using a stochastic gradient descent with a momentum of 0.9 that was optimised at an initial learning rate of 0.05. L2 regularisation was 0.0001. Minimum batch size was 16 images, and training data were shuffled at every epoch. Training took 3483 min and yielded a mini‐batch accuracy of 85.85% at the final iteration. Due to the small sample size, we did not perform rigorous testing for example, independent test set to for u‐net prediction robustness. However, machine leaning outputs were validated (and correlated well) with manual measurements.

To predict image subtypes for all OCT frames, we used the trained model followed by operations to remove isolated pixels and small islands. Boundaries between classes were smoothed with a Fourier descriptor. Due to imaging limitations associated with OCT penetration, we limited analysis to a skin depth of 1 mm, with 2D frame outputs in mm^2^ multiplied by the scanning interval (50 μm) for 3D scan outputs in mm^3^ (± standard deviation, S.D.). Automated annotation interobserver reliability was assessed for all 204 scans, representing 104 averaged biopsy samples.

### 3.5 Statistical methods

All data groups followed a normal distribution. Grouped analyses were performed using 95% confidence intervals and a two‐way analysis of variance mixed‐effects model with *post hoc* testing corrected for multiple comparisons using Sidak's test (GraphPad Prism, La Jolla, California). Correlations were analysed using Pearson's correlation testing (95% CI) and Spearman's rank for non‐parametric data.

## 4 RESULTS

### 4.1 Machine learning validation

**FIGURE 1:**
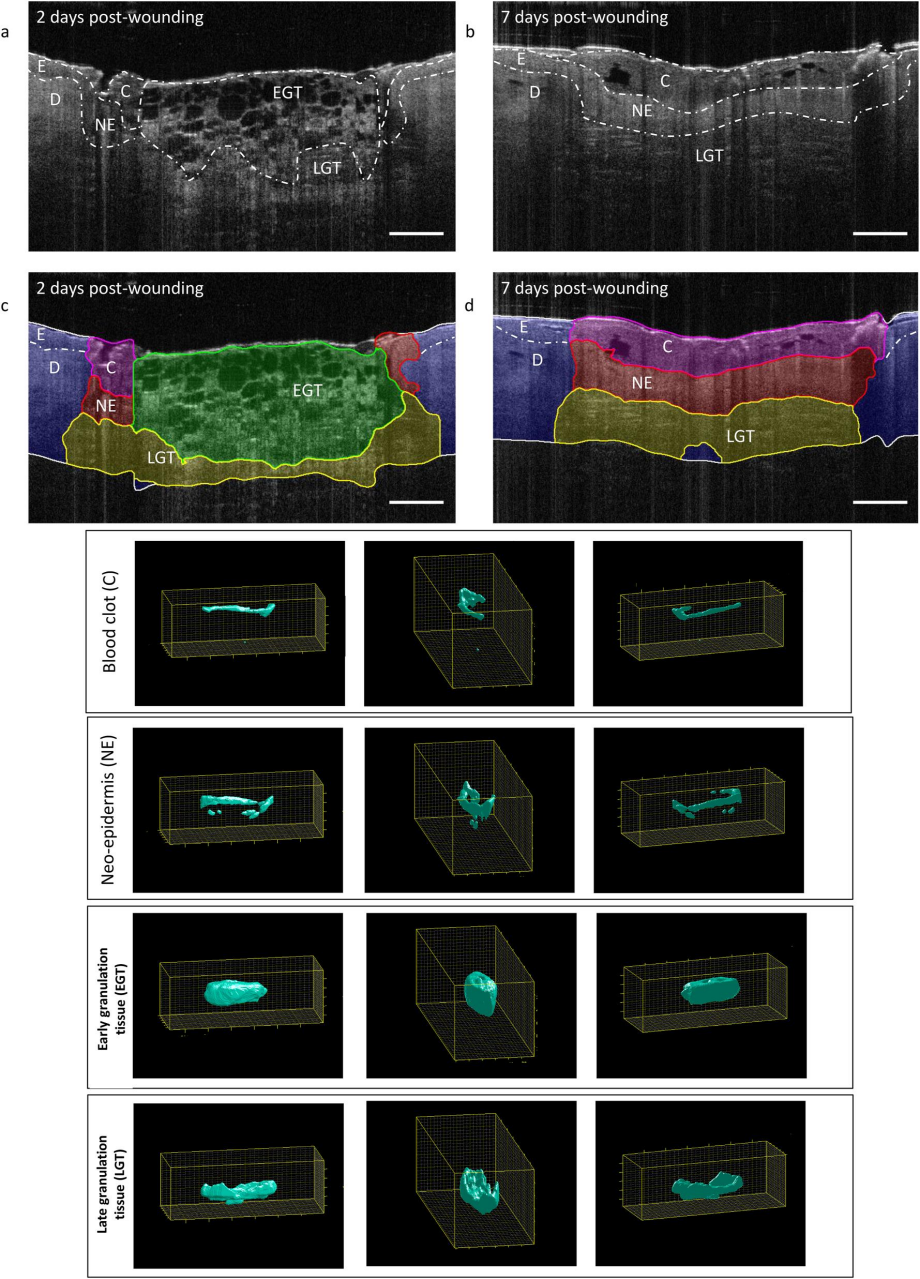
Machine learning wound healing segmentation. Representative original OCT single frame outputs (a, b), annotated machine learning OCT single frame outputs (c, d), and 3D OCT scan rendering. C, blood clot; D, dermis (intact tissue) at day 2 and seven post‐wounding; E, epidermis; EGT, early granulation tissue; LGT, late granulation tissue; NE, neo‐epidermis. Scale bar = 500 μm.

Wound morphology was classified into four key morphological features: (1) early granulation tissue; (2) late granulation tissue; (3) neo‐epidermis; and (4) blood clot. Illustrative examples of typical machine‐annotated OCT frame outputs and 3D renderings from days 2 and seven post‐wounding are presented in Figure 1 (and Supplemental Figure S1).

**FIGURE 2:**
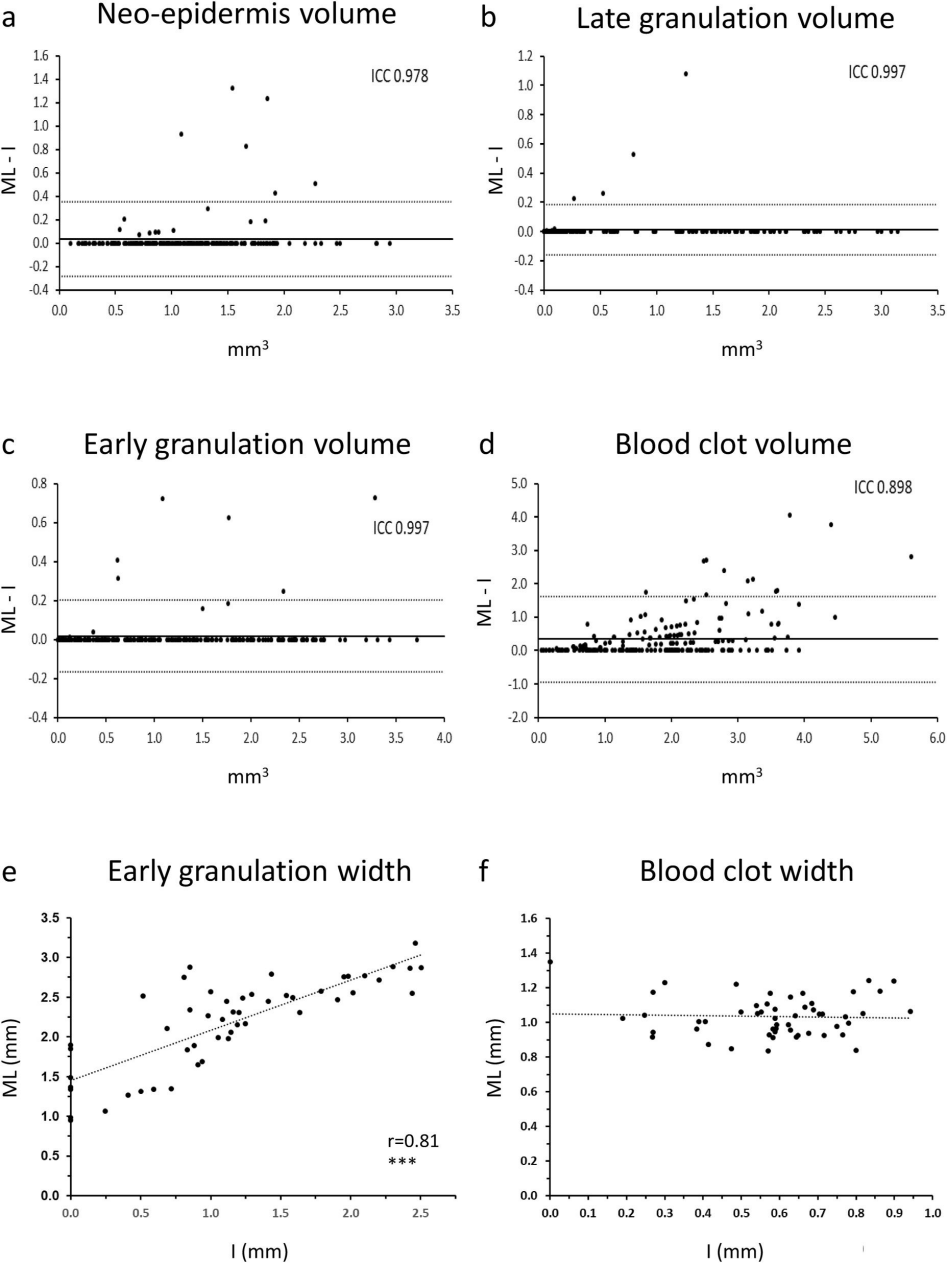
Machine learning reliability. Altman plots neo‐epidermis (a), late granulation tissue (b), early granulation tissue (c), and blood clot (d) are shown. Intraclass correlation coefficients (ICC) are displayed on each plot. Solid lines indicate the bias values, and dotted lines (+2 SD and −2 SD) indicate the 95% limit of agreement (*n* = 204). Correlations between automated measurement and manual measurement for early granulation tissue (e, *n* = 51) and blood clot depth (f, *n* = 50). I, investigator measurement; ML, machine learning measurement. Significance *** = *p* < 0.001.

Automated annotation accuracy was assessed for all scans. Investigator agreement with machine annotation accuracy (inter‐observer reliability) was excellent (ICC > 0.8) for all sub‐types with ICC >0.95 for early granulation, late granulation and neo‐epidermal tissue and ICC >0.85 for clot tissue (Figures 2a–2d).

We observed a strong correlation (*r* = 0.81, *p* < 0.001) between automated early granulation tissue width and corresponding manual measurements (Figure 2e). However, this was not observed for blood clot depth (Figure 2f).

### 4.2 Wound morphology

#### 4.2.1 Gross morphology and re‐epithelialisation

**FIGURE 3:**
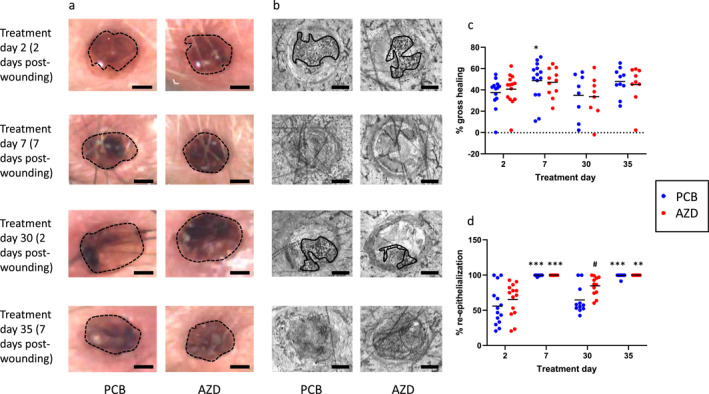
Gross morphology and re‐epithelialisation. Representative images for post‐wounding day 2 (treatment days 2 and 30) and day 7 (treatment days 7 and 35) treated with AZD or PCB for gross morphology (a) and re‐epithelialisation (b), quantified manually as % gross healing (c, PCB treatment day 2 [*n* = 13], day 7 [*n* = 14], day 30 [*n* = 8], day 35 [*n* = 10] and AZD treatment day 2 [*n* = 14], day 7 [*n* = 11], day 30 [*n* = 8], day 35 [*n* = 9]) and % re‐epithelialisation (d, PCB treatment day 2 [*n* = 14], day 7 [*n* = 14], day 30 [*n* = 11], day 35 [*n* = 13] and AZD treatment day 2 [*n* = 14], day 7 [*n* = 14], day 30 [*n* = 12], day 35 [*n* = 13]). * = day 2 wound versus day 7 wound (repeated at treatment day 28 vs. day 35), # = PCB versus AZD. Significance * = *p* < 0.05, ** = *p* < 0.01, *** = *p* < 0.001.

Gross wound area derived using conventional digital photography was comparable between AZD and PCB (Figure 3a). Using this indicator, healing improved from 37% to 49% between day 2 and day 7 with PCB (*p* < 0.05, Figure 3c) in biopsies conducted on day 0 (set 1) but this was not significant in repeat biopsies on treatment day 28 (set 2) or either set of biopsies with AZD. Similarly, no treatment effect was observed (Figure 3a,c).

By contrast, manual OCT image analysis found increased re‐epithelialisation (mean ± standard deviation) at day 7 versus day 2 post‐wounding with PCB and AZD (PCB set 1: 99.7 ± 0.8 vs. 56 ± 27, set 2: 99.3 ± 2.4 vs. 64.6 ± 19.7, both *p* < 0.001, and AZD set 1: 100 ± 0 vs. 65.3 ± 23.6, *p* < 0.001, set 2: 100 ± 0 vs. 84.7 ± 13.5, all *p* < 0.01, Figure 3b,d).

Further, at day 2 post‐wounding after 30 days of treatment, re‐epithelialisation by manual OCT was 30% greater with AZD (*p* < 0.05, Figure 3d). Re‐epithelialisation was complete in most cases by day 7 post‐wounding (Figure 3d).

#### 4.2.2 Automated volumetric analysis

**FIGURE 4:**
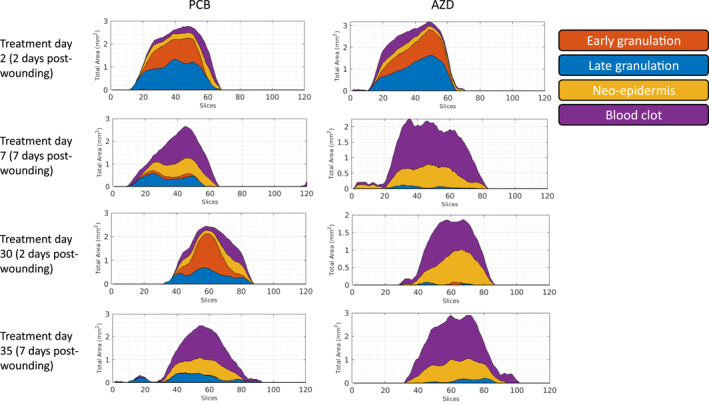
Machine‐learning histograms. Representative histograms for post‐wounding day 2 (treatment days 2 and 30) and day 7 (treatment days 7 and 35) treated with AZD or PCB. Day 2 wound morphology at treatment day 30 more closely resembles that of day 7 wounds for AZD, but not PCB.

Representative histograms displaying tissue subtype area for each OCT scan frame (120 frames per scan) are presented in Figure 4, indicating that day 2 wound morphology more closely resembles that of day 7 following 30‐day treatment with AZD.

**FIGURE 5:**
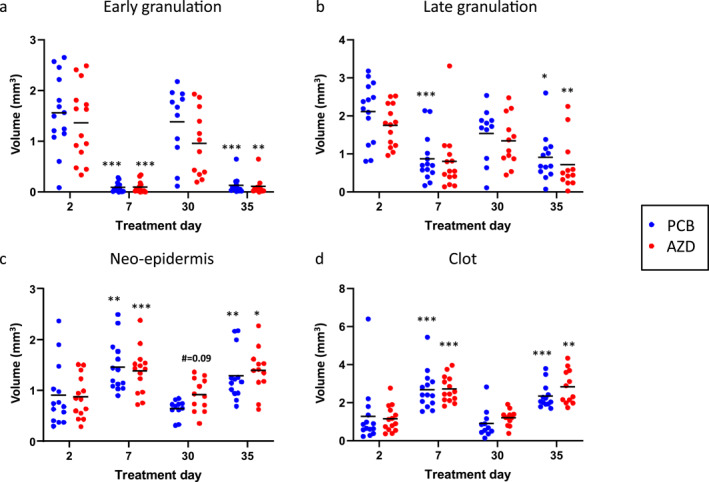
Machine learning volumetric quantification. Machine learning outputs for early (a) and late (b) granulation, neo‐epidermis (c), and blood clot (d) tissue for post‐wounding day 2 (treatment days 2 and 30) and day 7 (treatment days 7 and 35) treated with AZD or PCB. PCB treatment day 2 (*n* = 14), day 7 (*n* = 14), day 30 (*n* = 11), day 35 (*n* = 13) and AZD treatment day 2 (*n* = 14), day 7 (*n* = 14), day 30 (*n* = 12), day 35 (*n* = 12). * = day 2 wound versus day 7 wound (repeated at treatment day 28 vs. day 35). Significance * = *p* < 0.05, ** = *p* < 0.01, *** = *p* < 0.001.

Early granulation tissue volume was lower on day 7 versus day 2 post‐wounding with PCB and AZD in both sets of biopsies (PCB set 1: 0.09 ± 0.09 vs. 1.56 ± 0.7, set 2: 0.13 ± 0.17 vs. 1.38 ± 0.7, both *p* < 0.001 and AZD set 1: 0.1 ± 0.1 vs. 1.36 ± 0.7, *p* < 0.001, set 2: 0.11 ± 0.2 vs. 0.96 ± 0.6, *p* < 0.01, Figure 5a). At day 2 post‐wounding (treatment day 30), the effect with AZD was in the expected direction, but not statistically significant (*p* = 0.48, Figure 5a). The change was also in the expected direction but not statistically significant with AZD at day 2 post‐wounding and treatment day 2 (*p* = 0.93). At 7 days post‐wounding, resolution of early granulation tissue was largely complete.

Late granulation tissue volume was also lower on day 7 versus day 2 post‐wounding (PCB set 1: 0.87 ± 0.6 vs. 2.12 ± 0.8, *p* < 0.001, set 2: 0.91 ± 0.6 vs. 1.54 ± 0.7, *p* = 0.25 and AZD set 1: 0.81 ± 0.8 vs. 1.75 ± 0.5, *p* < 0.05, set 2: 0.72 ± 0.7 vs. 1.34 ± 0.7, *p* < 0.001, Figure 5b). At 2 days post‐wounding and treatment day 2, AZD had an effect in the expected direction, but this was not statistically significant (*p* = 0.53, Figure 5b).

By contrast, neo‐epidermis volume was higher in day 7 than day 2 wounds (PCB set 1: 1.46 ± 0.5 vs. 0.9 ± 0.6, *p* < 0.01, set 2: 1.29 ± 0.5 vs. 0.65 ± 0.2, *p* < 0.001 and AZD set 1: 1.38 ± 0.4 vs. 0.87 ± 0.4, *p* < 0.01, set 2: 1.39 ± 0.5 vs. 0.92 ± 0.3, *p* < 0.05, Figure 5c). At day 2 post‐wounding and treatment day 30, there was also a trend towards a 42% increase in neo‐epidermal volume by AZD (*p* = 0.09, Figure 5c). This was not apparent at day 2 post‐wounding and treatment day 2 (*p* = 0.99). At 7 days post‐wounding, re‐epithelialisation was mostly complete.

Similarly, blood clot volume was higher in day 7 than day 2 wounds (PCB set 1: 2.68 ± 1 vs. 1.28 ± 1.6, set 2: 2.35 ± 0.6 vs. 0.91 ± 0.7, both *p* < 0.001 and AZD set 1: 2.73 ± 0.7 vs. 1.16 ± 0.7, *p* < 0.001, set 2: 2.83 ± 0.9 vs. 1.21 ± 0.4, *p* < 0.01, Figure 5d), consistent with a more advanced stage of healing. At day 7 post‐wounding, after 7 and 35 days of treatment, the change in clot volume with AZD was in the expected direction but not statistically significant (*p* = 0.99 and *p* = 0.45, respectively, Figure 5d).

#### 4.2.3 Machine learning agreement with wound re‐epithelialisation

Further to the correlation between manual and automated early granulation tissue width (Figure 2e), all four automated wound morphology volumes correlated well with wound re‐epithelialisation by manual OCT image analysis (early granulation *ρ* = −0.90, late granulation *ρ* = −0.73, neo‐epidermis *ρ* = 0.66 and clot *ρ* = 0.71, all *p* < 0.001, Supplemental Figure S2).

To further assess algorithm robustness, we also analysed all available unwounded (baseline and treatment day 35) and day 30 post‐wounding (fully healed) OCT scans. As anticipated, detection of wound morphologies in these samples were negligible (Supplemental Figure S3).

Representative, annotated OCT scans for each treatment and time point are presented in Supplemental Figure S4.

## 5 DISCUSSION

To our knowledge, this is the first use of machine learning to automate volumetric OCT wound tissue subtypes, representing a significant advance in OCT development as a wound research tool. OCT is a validated, noninvasive method for monitoring skin wound healing though ‘virtual biopsy’,^21^, ^22^, ^23^, ^24^, ^25^ but previous studies were limited by subjective and labour‐intensive manual annotation. Here, we present key advantages over conventional digital photography or single‐frame OCT, including higher sensitivity, objectiveness, and rapid wound morphology multiplex analysis that would not be feasible to conduct manually.

Despite relatively complex and dynamic OCT scans, algorithm reliability (i.e., investigator agreement with machine annotation) was excellent for all wound morphologies, with negligible detection in unwounded and healed tissue. Further, algorithm outputs were validated against manual measurements from our randomized, controlled trial,^13^ with a strong correlation for early granulation tissue width. However, this was not observed for clot depth, possibly due to lower machine learning sensitivity (more heterogeneous morphology) and overall data variability for this wound feature.

Importantly, machine learning outputs also corroborated our finding of improved wound healing in patients with type 2 diabetes following oral 11β‐HSD1 inhibition with AZD,^13^ with 30% greater re‐epithelialisation and corresponding trend in reduced neo‐epidermis volume. These results are supported by mechanistic findings that glucocorticoids impair epidermal re‐epithelialisation by suppressing keratinocyte growth factor signalling.^9^, ^10^ However, a limitation of this pilot clinical trial was the exclusion of patients with active ulcers, pending initial healing outcomes in an acute wound setting. Therefore, healing was relatively normal (potentially limiting perceived AZD effectiveness), supported by our in vivo studies in young, healthy mice.^31^

Although loss of 11β‐HSD1 inhibitor efficacy has been reported in some tissues for example, adipose (but not liver) following 2 weeks' treatment in healthy volunteers,^32^ this was not apparent for AZD in skin. Indeed, improved wound healing was more pronounced at treatment day 28 versus treatment day 2,^13^ upheld by machine learning results presented here. Loss of 11β‐HSD1 inhibitor efficacy could depend on various factors (e.g., compound composition, dose and formulation, target tissue, disease, and patient demographics). Demonstrating 11β‐HSD1 inhibitor efficacy at the tissue level and identifying patients most likely to require and respond to 11β‐HSD1 inhibitor therapy are important areas of ongoing research.

Although beyond the scope of the current study, algorithm development in chronic ulcers could identify novel predictive biomarkers of healing for more targeted patient management. Such biomarkers could also improve stratification and robustness of future clinical trials.

## CONFLICTS OF INTEREST

The authors state no competing financial interests. The content of this article was expressly written by the authors listed. No ghostwriters were used to write this article.

## AUTHOR CONTRIBUTIONS

**Yinhai Wang**: Data curation (Lead); Formal analysis (Equal); Investigation (Lead); Methodology (Lead); Resources (Equal); Software (Lead); Validation (Equal); Visualization (Equal); Writing – review & editing (Equal). **Adrian Freeman**: Conceptualization (Equal); Resources (Equal); Supervision (Equal); Writing – review & editing (Equal). **Ramzi Ajjan**: Resources (Supporting); Supervision (Supporting); Writing – review & editing (Supporting). **Francesco Del Galdo**: Conceptualization (Supporting); Investigation (Supporting); Resources (Supporting); Supervision (Equal); Writing – review & editing (Equal). **Ana Tiganescu**: Conceptualization (Equal); Formal analysis (Equal); Funding acquisition (Lead); Investigation (Supporting); Project administration (Lead); Resources (Equal); Supervision (Equal); Validation (Equal); Visualization (Equal); Writing – original draft (Lead); Writing – review & editing (Equal).

## ETHICS STATEMENT

This study was conducted with approval from the North West Greater Manchester Central Research Ethics Committee (17/NW/0283), with study participant written informed consent.

## Supporting information

Figure S1

Figure S2

Figure S3

Figure S4

## Data Availability

Source code is available on GitHub at https://github.com/AstraZeneca/OCT_publication, with the code for implementation of U‐net architecture within it. The trained U‐net model used in this study is also hosted in this repository with example images for testing purposes.
