# Supplementary material for: Automated quantification of 3D wound morphology by machine learning and optical coherence tomography in type 2 diabetes
Source: Skin Health Dis. 2022 Dec 21;3(3):e203. doi: 10.1002/ski2.203 (PMC10233090; doi:10.1002/ski2.203)

Supplemental Figure S1. Unannotated representative OCT scan frames for day 2 (a) and day 7 (b) wounds in Figure 1.

a)

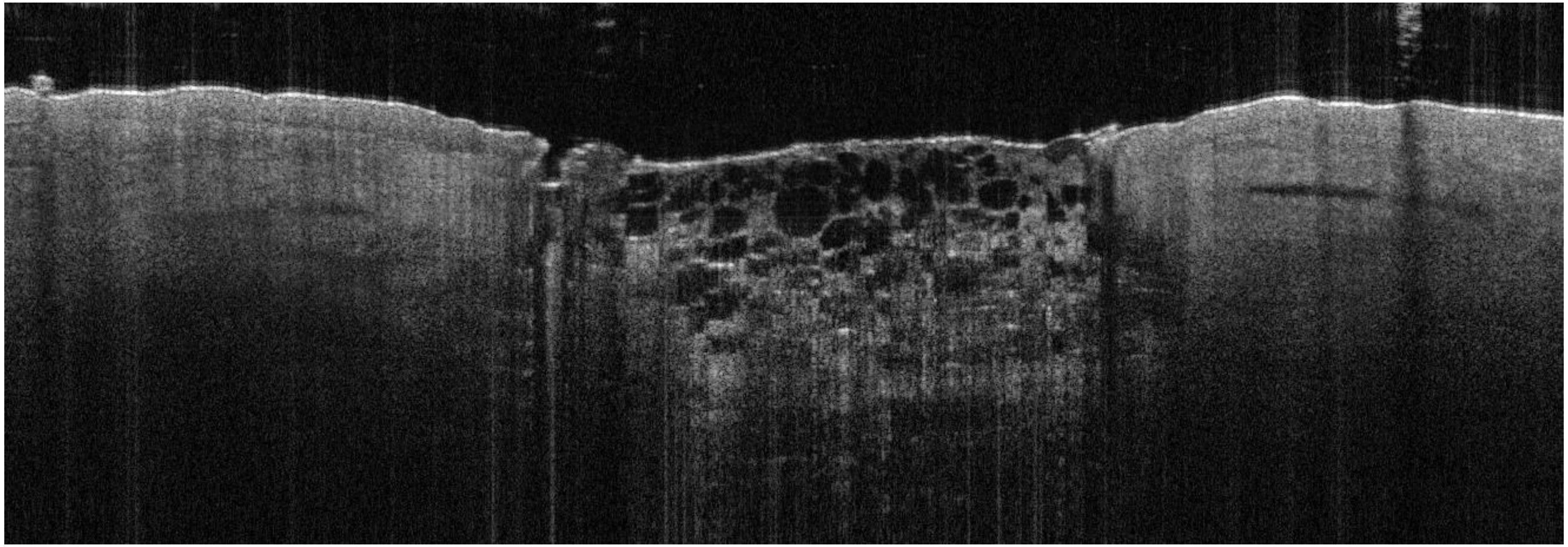

b)

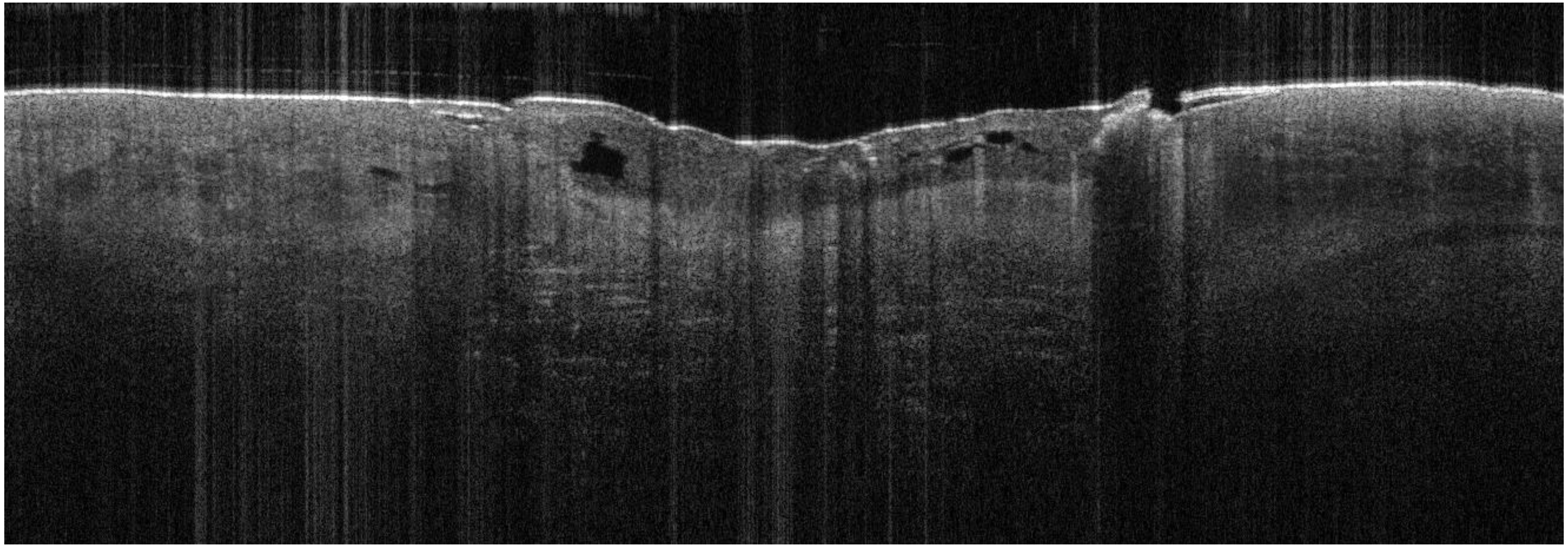

Supplement: Supplementary file 1 — Figure S1 [file SKI2-3-e203-s004.pdf]
