# Supplementary material for: Automated quantification of 3D wound morphology by machine learning and optical coherence tomography in type 2 diabetes
Source: Skin Health Dis. 2022 Dec 21;3(3):e203. doi: 10.1002/ski2.203 (PMC10233090; doi:10.1002/ski2.203)

Supplemental Figure S2. Spearman's correlations between machine learning outputs (ML) and investigator measurement of re-epithelialization, as in Figure 3d, (I, n=100-104). Significance *** = p<0.001

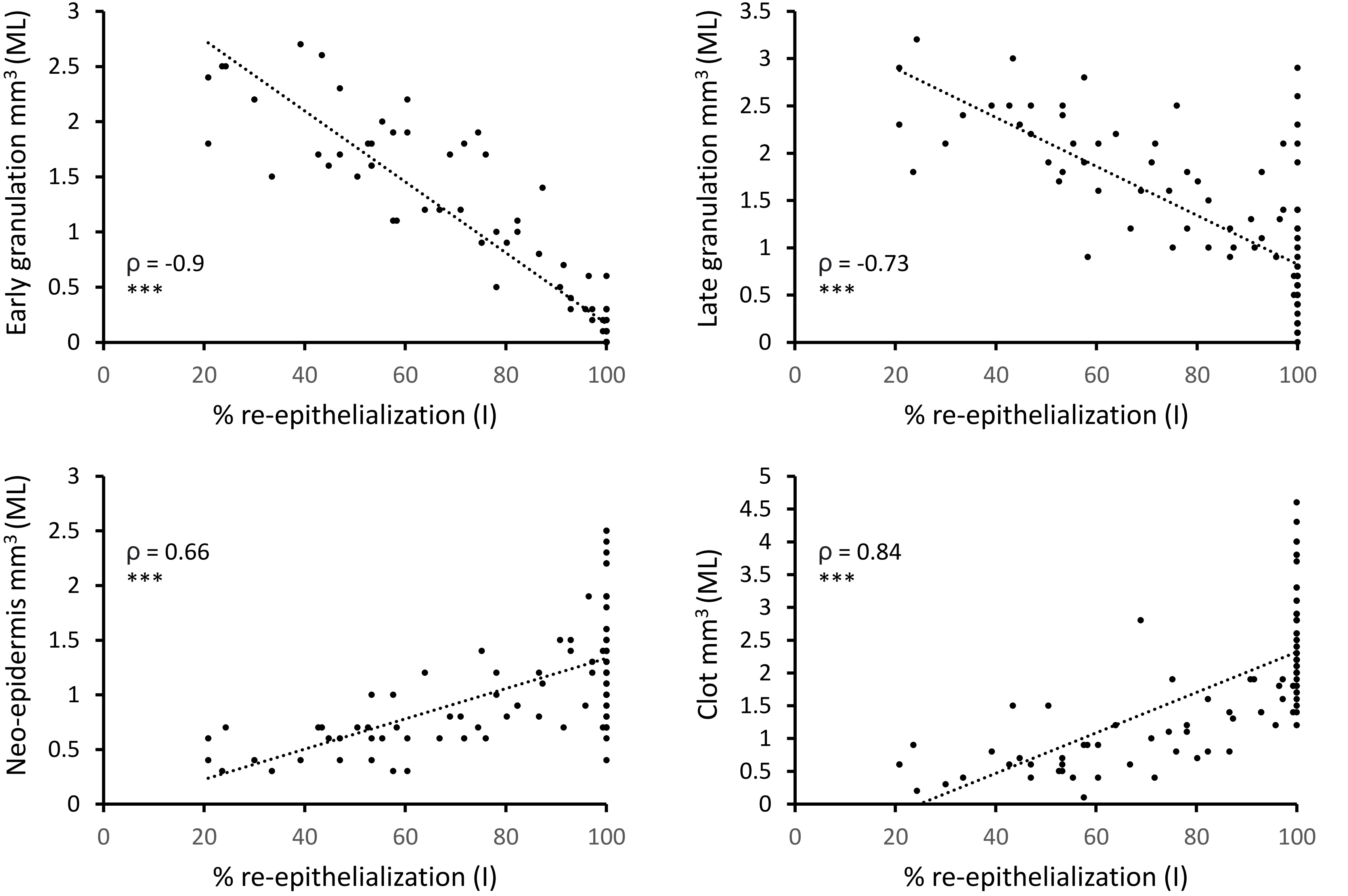

Supplement: Supplementary file 2 — Figure S2 [file SKI2-3-e203-s002.pdf]
