# Supplementary material for: Automated quantification of 3D wound morphology by machine learning and optical coherence tomography in type 2 diabetes
Source: Skin Health Dis. 2022 Dec 21;3(3):e203. doi: 10.1002/ski2.203 (PMC10233090; doi:10.1002/ski2.203)

Supplemental Figure S3. Machine learning outputs ± S.E for baseline and day 35 unwounded skin (adjacent) and day 30 post-wounding. AZD4017 (AZD), placebo (PCB). PCB baseline (n = 14), day 30 wound (n = 10), day 35 unwounded (n = 13), day 35 (n = 13) and AZD baseline (n = 14), day 30 wound (n = 12), day 35 unwounded (n = 12). Y-axis scales match Figure 5

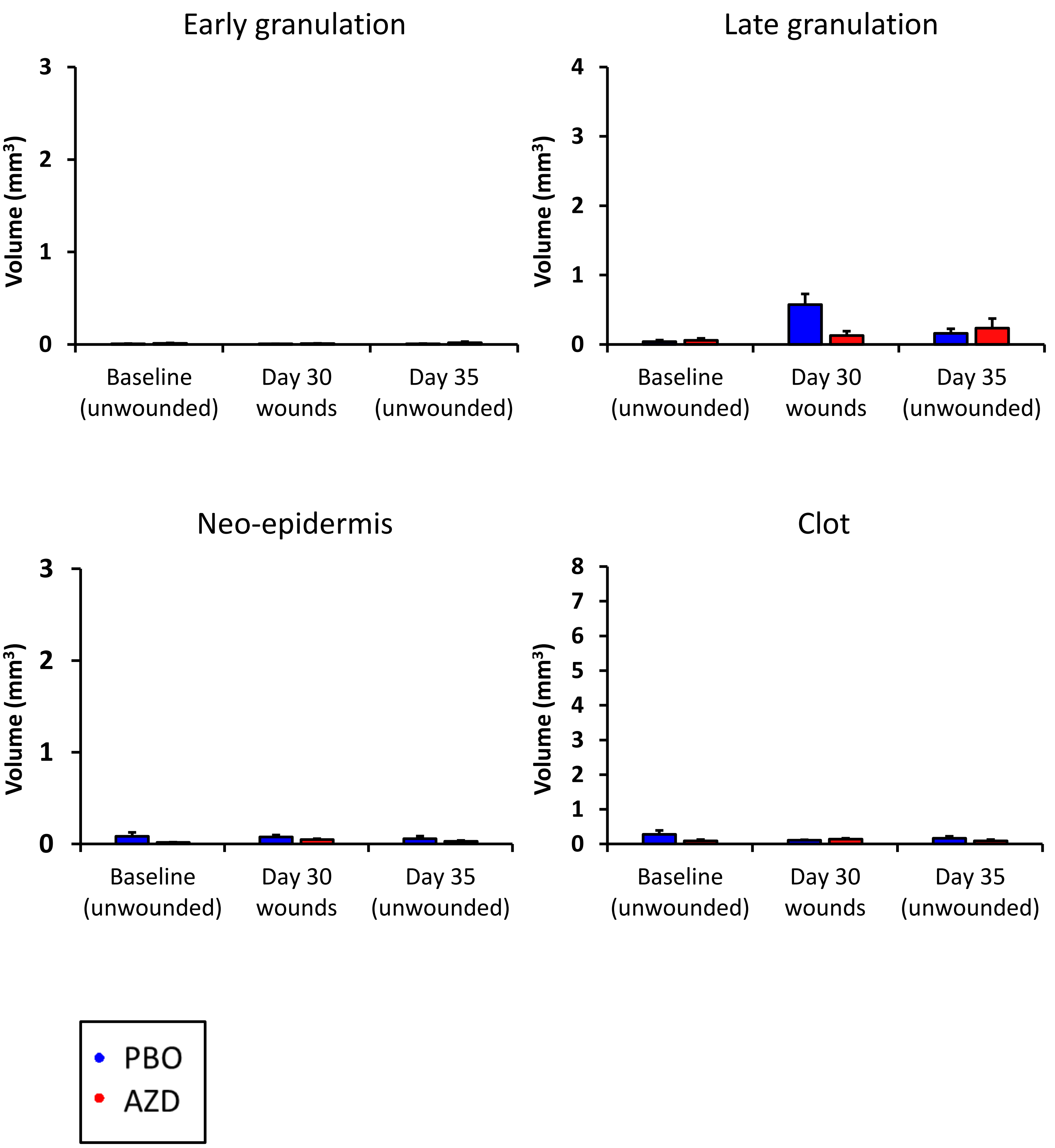

Supplement: Supplementary file 3 — Figure S3 [file SKI2-3-e203-s003.pdf]
