# Supplementary material for: Automated quantification of 3D wound morphology by machine learning and optical coherence tomography in type 2 diabetes
Source: Skin Health Dis. 2022 Dec 21;3(3):e203. doi: 10.1002/ski2.203 (PMC10233090; doi:10.1002/ski2.203)
Supplement: Supplementary file 4 — Figure S4 [file SKI2-3-e203-s001.pdf]

Supplemental Figure S4. Representative OCT scans annotated by machine learning at post-wounding day 2 / treatment day 2 with placebo (PCB, A) or AZD4017 (AZD, B), post-wounding day 7 / treatment day 7 with PCB, (C) or AZD (D), post-wounding day 2 / treatment day 30 with PCB (E) or AZD (F), and post-wounding day 7 / treatment day 35 with PCB (G) or AZD (H). Negligible OCT annotation was observed with day 0 (baseline) adjacent unwounded skin (I), post-wounding day 30 / treatment day 30 with PCB (J) or AZD (K), or treatment day 35 adjacent unwounded skin (L). See also Figures 5 and S4.

A) PCB treatment day 2 wound day 2

B) PCB treatment day 7 wound day 7

C) PCB treatment day 30 wound day 2

D) PCB treatment day 35 wound day 7

E) AZD treatment day 2 wound day 2

F) AZD treatment day 7 wound day 7

G) AZD treatment day 30 wound day 2

H) AZD treatment day 35 wound day 7

I) day 0 unwounded

J) PCB treatment day 30 wound day 30

K) AZD treatment day 30 wound day 30

L) day 35 unwounded
